# A two-step approach for the analysis of hybrids in comparative social policy analysis: a nuanced typology of childcare between policies and regimes

**DOI:** 10.1007/s11135-016-0423-1

**Published:** 2016-10-13

**Authors:** Rossella Ciccia

**Affiliations:** School of Social Sciences, Education and Social Work, Queen’s University Belfast, 6 College Park, Belfast, BT7 1NN United Kingdom

**Keywords:** Fuzzy set ideal type analysis, Hybrids, typology, Welfare regime, Child care, Gender equality, Comparative social policy

## Abstract

Typologies have represented an important tool for the development of comparative social policy research and continue to be widely used in spite of growing criticism of their ability to capture the complexity of welfare states and their internal heterogeneity. In particular, debates have focused on the presence of hybrid cases and the existence of distinct cross-national pattern of variation across areas of social policy. There is growing awareness around these issues, but empirical research often still relies on methodologies aimed at classifying countries in a limited number of unambiguous types. This article proposes a two-step approach based on fuzzy-set ideal type analysis for the systematic analysis of hybrids at the level of both policies (step 1) and policy configurations or combinations of policies (step 2). This approach is demonstrated by using the case of childcare policies in European economies. In the first step, parental leave policies are analysed using three methods—direct, indirect, and combinatory—to identify and describe specific hybrid forms at the level of policy analysis. In the second step, the analysis moves on to investigate the relationship between parental leave and childcare services. Clearly shows that many countries display characteristics normally associated with different types (hybrids and sub-types) . Therefore, this two-step approach demonstrates that disaggregated and aggregated analyses are equally important to account for hybrid welfare forms and make sense of the tensions and incongruences within and between policies.

## Introduction

Typologies have been central to the development of the field of comparative social policy. Ever since the publication of Esping-Andersen *Three Worlds of Welfare Capitalism* (1990) classifying countries in three distinct welfare regime types, the use of regime typologies to group countries and capture cross-national variation in social policy organization has been booming. There is an ongoing debate in the literature over the actual number of types, the particular classification of certain countries and many have tried to come up with alternative typologies. Feminist scholars have also engaged in this endeavour, producing many typologies of childcare and family policies (Crompton 1999; Lewis 1992; Orloff 1993; Sainsbury 1996). These contributions have significantly advanced our knowledge of the different ways in which social institutions and policies interact to produce specific patterns of inequalities. Nonetheless, the enduring usefulness of this kind of analysis is increasingly put under scrutiny. There is growing consensus that welfare states are likely to exhibit hybrid forms showing features of more than one type. Many welfare states (e.g. Belgium, Ireland, the Netherlands, and Switzerland) are notoriously hard to classify, and even countries (e.g. the United Kingdom) generally considered as prototype differ from this on important aspects (e.g. universal healthcare). While in theory the existence of hybrids is well-recognized, this is only partly reflected in research practices which too often still rely on concepts and methodologies based on the idea of unambiguous types.

The problem of how to analyse hybrid welfare states is receiving growing attention in comparative welfare state literature. From a conceptual viewpoint, many argue the need for disaggregated policy-centred approaches in order to highlight potential incongruences and diverse policy histories and actors (Bannink and Hoogenboom 2007; Blofield and Franzoni 2015; Kasza 2002; Wincott 2006). Others emphasize that our ability to adequately describe the internal heterogeneity of national welfare states depends also crucially on the choice of method (Hudson and Kuhner 2010). In this view, fuzzy set ideal type analysis has increasingly found application in comparative social policy studies to avoid top-down simplification, grasp welfare state complexity and possibly detect small changes signalling contradictory policy directions (Lee 2011). In particular, this method allows to map differences and changes in social policies without being constrained by the idea that welfare states must necessarily represent packages of congruent arrangements. Nonetheless, these studies have also generally focused on the (partial) membership of countries in particular types, rather than investigating the portion which they fit poorly or describing the plurality of forms that are found within them.

This article contributes to the literature on hybrids and welfare state typologies by proposing a two-step approach based on fuzzy-set ideal type analysis (hereafter FSITA) for the systematic analysis of hybrids in comparative social policy analyses. This approach maintains the importance of the analysis of both single and combinations of policies in order to systematically understand the nature of welfare state hybridity. The paper is structured as follows. First, it begins by discussing debates about the choice of the level of analysis. Next, it illustrates the potential of FSITA in dealing with hybrid cases, and presents the theoretical model informing the subsequent analyses illustrating the two-step approach. This procedure is demonstrated by drawing on the results of analyses of parental leave and early childhood and education (ECEC) services. The first step focuses on parental leave regulations to demonstrate three methods—direct, indirect, combinatory—to detect and characterize hybrids at the level of policy analysis. The second step moves on to aggregate the results of analyses of parental leave and ECEC provisions in order to develop an overall typology, which clearly shows the co-existence of multiple types in many countries’ childcare policies. This also serves to illustrate that based on the relationship between policies, it is possible to distinguish different types of configurations—pure, hybrid and sub-types.

## Regimes or policies? Moving beyond a false dilemma

Few concepts are at the same time as popular and debated as that of welfare regime in comparative social policy. Debates have focused on many issues (see Arts and Gelissen 2002), but recently a more radical critique has questioned the usefulness of regime typologies versus more disaggregated, policy-centred analysis (Bannink and Hoogenboom 2007; Blofield and Franzoni 2015; Hinrichs 2000; Kasza 2002; Wincott 2006). Three fundamental limitations of regime analysis are identified: complexity within policies, complexity between policies and the ability to account for processes of welfare state change.

First, policies are multifaceted objects and even small details have considerable consequences on the way we understand and classify them. Studies adopting a policy approach to study parental leave (Haas 2003) and childcare services (Wincott 2006) show that, ‘if god is in details anywhere, it is surely in work-family policy design’ (Gornick and Meyers 2009). The matter is further complicated by the fact that policies reflect multiple, potentially contradictory goals. For instance, childcare policies generally aim at some combination of the following objectives: increasing fertility, reducing poverty, promoting child’s development, encouraging maternal employment and diminishing gender inequalities. Consequently, this field remains characterized by tensions over the treatment of non-parental care, the responsibilities of the state and private actors and the regulation of the social care market (Morgan 2013). Given these complexities, an approach focusing on the characteristics of policies can provide more accurate descriptions of their functioning and underlying principles.

The second reason to disaggregate welfare regimes concerns the relationship between policies. Welfare states often adopt different approaches to different social risks and even the approach to each social risk is often hybrid (Wincott 2006). For instance, public support for childcare and elderly care are often quite different within a single state and subject to different dynamics of expansion/retrenchment (Anttonen and Sipilä 1996). This points to the possibility of inconsistencies in the relationship between social policies, which tend to remain concealed in regime typologies. In this view, a policy approach can also contribute to making sense of the various ways in which policies interconnect within different welfare arrangements.

Finally, it is argued that the idea of regimes, emphasising the overall stability of welfare systems, distorts our ability to understand processes of change. Reform dynamics are likely to differ not only between countries but also across fields of policy. For instance, family policies is an area of general expansion in advanced welfare states, in spite of retrenchment in other areas such as unemployment insurance (Ferragina et al. 2012). Each social policy appears to have its own conditions of development and different constellation of actors both supporting and implementing them (Kasza 2002). Accordingly, a disaggregated approach is also better suited to understand processes of welfare state change (Hinrichs 2000).

Given these reasons, some authors have remained sceptical of the possibility of identifying coherent welfare arrangements or even of devising typologies of single policies (Kasza 2002). Nonetheless, the idea of regimes cannot be easily dismissed in spite of its limitations. An important insight of regime analysis is that policy outcomes and usages are shaped by bundles of ‘inseparable provisions’ and not by single policies (Gornick and Meyers 2003). Moreover, each policy can generate countervailing effects and tradeoffs or have functional equivalents (Pettit and Hook 2012). Therefore, a comprehensive account of how social policies shape social outcomes requires both policy and regime analysis, but with a clear focus on the articulation of policies and the ways they interact within different welfare arrangements. Indeed, one of the aim of this paper is to show the usefulness of a two-step approach moving from policies to policy configurations in order to account for the presence of hybrid welfare forms and make sense of tensions and contradictions within and between policies.

## Fuzzy set ideal type analysis and hybrids

Our ability to deal with hybrid cases depends also crucially on the choice of a method for constructing typologies. In particular, the presence of cases with features of more than one type represent a problem for inductive approaches (e.g. relative indices, cluster analysis), which aim at synthetizing empirical patterns of variation and allocating cases to only one type. Since hybrids risk to invalidate these classifications, they are usually conceptualized as outliers, something to deal with or remove. Differently, hybrids do not necessarily imply a refutation of the typology for deductive approaches such as FSITA. FSITA has its origin in Qualitative Comparative Analysis (QCA) and fuzzy-set social science (Bellman and Zadeh 1970; Ragin 2000) and is a method to categorize cases based on theoretical concepts (Kvist 2007). FSITA starts from a concept of theoretical interest, translates it into sets or dimensions, which combine into a number of configurations (or ideal types), and then uses fuzzy set principles to compute memberships in those configurations (Vis 2007). These ideal types are theoretical abstractions constructed for their heuristic function with no empirical reality, and thus need not to be mutually exclusive. This is apparent in the use of partial memberships (0.5 < fuzzy score < 1) in FSITA to measure the distance/proximity of cases from any given type. Previous studies have used this feature to allocate cases to types in a more graduated manner, but have also generally continued to focus on membership (albeit partial). In step 1 below, this paper shows how the study of non-membership scores and the dimensions underlying the configurational space can be used to identify hybrid cases and the elements of other models which they also present.

Another feature of FSITA is also useful to make sense of potential incongruences in the relationship between policies and between policy dimensions. At the core of this method is a holistic approach to cases, the way in which attributes combine is just as important as their value. This is reflected in the use of the principle of set intersection (or logical AND) to compute membership scores, i.e. for a case to be a member of a configuration, it has to achieve a minimum score (fuzzy score >0.50) on all its constituting dimensions. Theory and the interpretation of cases play an important role in this process since thresholds to establish membership and the configurations themselves are defined based on theoretical knowledge. This principle enhances our ability to provide substantial interpretation to all the relevant policy combinations, also those that are apparently contradictory, thus moving beyond the idea of welfare states as packages of congruent arrangements. This feature is particularly useful in making sense of the relationship between different policy measures as will be shown in step 2.

## Childcare policies and gender equality: the ideal types

Given the emphasis of FSITA on theory-driven measurement, it is first necessary to briefly introduce the theoretical models that inform the subsequent analysis of childcare policies before demonstrating a two-step approach for the study of hybrids. Childcare policy is an area of increased interest among scholars and policy makers alike, and has been the object of numerous typologies. The ideal types used here are drawn closely from Nancy Fraser’s (1994) work on alternative arrangements to the male breadwinner gender division of labour, particularly on her universal caregiver model, an utopian model that advances gender equality as something that should include not just women’s labour market participation, but also crucially men and care work. In spite of women’s increased employment everywhere, the division of unpaid work is still asymmetric with women performing the bulk of housework and childcare. Fraser’s model has had considerable influence on feminist analyses because it explicitly addresses this unbalance in the division of labour, which is generally considered the main source of gender inequality in social policy literature. Drawing on Fraser’s work, four main ideal type are identified based on their views on men’s and women’s engagement in paid and care work and women’s financial autonomy. Four further variants reflect either developments in incentives for women’s economic activity (e.g. one-and-a-half breadwinner) or differences in the role of the state (supported and unsupported universal breadwinner). These are variants because they do not hold different ideals about gender roles from their main type. The ideal types are the following:

1. *Male breadwinner* (MB): Men are mainly responsible for paid work, while women are responsible for childcare and other unpaid work and depend financially from their husbands. The *one*-*and*-*a*-*half breadwinner* model with women working part-time represents a modern variant of this model (Crompton 1999).
2. *Caregiver parity*: also maintains traditional gender roles, but the state compensates women for their unpaid work through allowances and other benefits linked to their caring role (maternalism).
3. *Universal breadwinner*: promotes the equal engagement of men and women in paid employment, but does not address unbalances in the division of care work. This type may (*supported universal breadwinner*) or may not (*unsupported universal breadwinner*) provide public supports for childcare (e.g. cash allowances, leave rights, childcare services).
4. *Universal caregiver*: represents Fraser’s gender equality ideal, which promotes the equal engagement of men and women in paid and care work. This type acknowledges and values individuals’ right to time to perform care activities, which is considered equal to the right to access paid employment. Nonetheless, childcare is considered a societal responsibility that must be supported and shared among a range of actors (e.g. men, women, state, employers). The *limited universal caregiver* is a variant more focused on employment outcomes.

While these models describe normative ideals, feminist welfare state scholarship has demonstrated that these ideals are also translated into policies (Crompton 1999; Lewis 1992). Childcare policy design reflects (multiple) preferred ideals over the best place for the care of children and men’s and women’s contributions, which have a direct influence in shaping parental choices over childcare. These works also emphasize that policy usages and outcomes are not shaped by single measures but by complex packages and policy interactions. Table 1 synthetizes the main features of parental leave and ECEC services policy design under the various ideal types.

**Table 1:** Main characteristics of ideal types of parental leave and ECEC policies

Model	Leave policies	ECEC services	Main aims and underlying principles
Male breadwinner (MB)	– The main beneficiaries are women – Long duration (3 years or more) – Unpaid or poorly paid – No incentives for fathers	– Low childcare coverage – Full-time or part-time services – Low public financial investment in services	– Promoting childcare in the home by the mother
One-and-a-half breadwinner (OHB)	– The main beneficiaries are women – Long duration (3 years or more) – Unpaid or poorly paid – No incentives for fathers	– High childcare coverage – Part-time services – Low public financial investment in services	– Promoting childcare in the home by the mother – Supporting women as secondary-earner
Caregiver parity (CGP)	– The main beneficiaries are women – Long duration (3 years or more) – Well-paid – No incentives for fathers	– Low childcare coverage – Full-time services – Low or moderate public financial investment in services	– Promoting childcare in the home by the mother – Rewarding mothers
Unsupported universal breadwinner (UUB)	– Women are the main beneficiaries – Duration <1 year – Unpaid or poorly paid – No incentives for fathers	– High childcare coverage – Full-time services – Low public financial investment	– Laissez-faire approach which indirectly reinforces gender inequalities in paid and unpaid work
Supported universal breadwinner (SUB)	– Women are the main beneficiary – Duration <1 year – Well-paid – No incentives for fathers	– High childcare coverage – Full-time services – High public financial investment	– Promoting labour market participation of men and women
Limited universal caregiver (LUC)	– Men and women have similar entitlements – Duration of around 1 year – Flexibility in terms of part-time and piecemeal leave – Well-paid – Incentives for men to take leave (e.g. daddy quota)	– High coverage – Full-time services – High public financial investment	– Promoting labour market participation of men and women – Increasing men’s involvement in childcare
Universal caregiver (UC)	– Men and women have similar entitlements – Longer duration – Flexibility in terms of part-time and piecemeal leave – Well-paid – Incentives for men to take leave (e.g. daddy quota)	– High coverage – Part-time services – High public financial investment	– Transforming gender roles for men and women – Increasing men’s involvement in childcare – Recognizing the individual right to time for childcare

## Step 1: analysing hybrids at the level of policy analysis

In what follows, I use the results of Ciccia and Verloo’s (2012) analysis of parental leave policies to demonstrate three methods—the direct, indirect and combinatory—to study hybrids at level of policy analysis. In their study, the authors classify parental leave policies in thirty European countries. The authors used four dimensions—duration (T), monetary compensation (M), distribution of entitlements (G), and incentives for fathers (F)—to translate Frasers’ models in six ideal typical policy configurations. In order to assign cases to types, each dimension was then transformed into a set by: (1) choosing an empirical indicator (operationalization); and (2) using substantive and theoretical knowledge to establish three breakpoints, full membership (1), no membership (0) and the crossover point (0.50) (calibration). The calibration of sets is a crucial task since it transforms empirical values into fuzzy scores (measures, thresholds and fuzzy scores are reported in Appendix 1, tables 7 and 8) Each ideal type corresponds to a particular combination of sets or configuration, i.e. a particualar corner of the property space descibed by all logically possible combination of dimensions (Appendix 1, Table 9). Set intersection (or the minimum fuzzy score value among the sets involved) was then used to establish memberhip (fs >0.50) and non membership (fs <0.50) scores indicating the extent to which a case is well-described by a particular ideal type. Table 2 shows the resulting membership scores of countries in those different types (see Ciccia and Verloo 2012 for broader discussion of models and findings).

**Table 2:** Fuzzy membership scores of parental leave policies, 2012

Country	Full universal caregiver (FUC)	Limited universal caregiver (LUC)	Supported universal breadwinner (SUP)	Unsupported universal breadwinner (UUB)	Caregiver Parity (CGP)	Male breadwinner (MB)
Austria	0.05	0.05	0.05	0.27	0.05	0.73
Belgium	0.16	0.21	0.21	**0.60**	0.16	0.16
Bulgaria	0.06	0.06	0.25	0.17	0.75	0.17
Cyprus	0.01	0.01	0.01	0.83	0.01	0.17
Czech Republic	0.02	0.00	0.00	0.00	0.00	0.94
Denmark	0.12	0.12	0.73	0.05	0.23	0.05
Estonia	0.05	0.03	0.03	0.03	0.18	0.79
European Union	0.00	0.00	0.00	0.80	0.00	0.20
Finland	0.22	**0.56**	0.05	0.05	0.00	0.05
France	0.00	0.00	0.00	0.00	0.01	0.87
Germany	0.01	0.01	0.01	0.05	0.01	0.03
Greece	0.03	0.03	0.03	0.80	0.03	0.20
Hungary	0.07	0.04	0.04	0.04	0.35	0.65
Iceland	0.14	0.86	0.01	0.01	0.01	0.01
Ireland	0.03	0.03	0.03	**0.59**	0.03	0.41
Italy	0.06	0.06	0.14	**0.60**	0.14	0.40
Latvia	0.01	0.01	0.01	0.02	0.01	0.66
Lithuania	0.26	0.03	0.03	0.03	**0.58**	0.33
Luxembourg	0.09	0.09	0.33	0.29	0.33	0.29
Malta	0.02	0.02	0.02	0.85	0.02	0.15
Netherlands	0.11	0.11	0.11	0.61	0.11	0.39
Norway	0.20	0.80	0.00	0.00	0.00	0.00
Poland	0.00	0.00	0.00	0.02	0.00	0.91
Portugal	0.22	**0.53**	0.00	0.00	0.00	0.00
Romania	0.07	0.07	0.15	0.08	0.84	0.08
Slovakia	0.03	0.03	0.03	0.04	0.03	0.84
Slovenia	0.15	0.15	0.65	0.09	0.35	0.09
Spain	0.00	0.00	0.00	0.00	0.00	0.79
Sweden	0.41	**0.59**	0.00	0.00	0.00	0.00
Switzerland	0.05	0.05	0.88	0.12	0.05	0.05
United Kingdom	0.00	0.00	0.00	0.48	0.00	**0.52**

Scores in bold indicate cases with very weak membership scores (0.50–0.60)

Adapted from Ciccia and Verloo (2012)

### Direct approach

The direct approach relies on the analysis of non-membership scores to identify and characterize hybrids. By inspecting countries’ membership scores, we are able to identify nine countries (Belgium, Finland, Ireland, Italy, Lithuania, the Netherlands, Sweden, Portugal and the United Kingdom) with low scores (0.50–0.60) (Table 2). In all these cases, policies are prevalently of one type, but also display inconsistencies and characteristics normally associated with other type policies. In order to identify those types, the next step is to examine their scores in the *non*-*membership configurations* (<0.50). In five of those cases, membership scores sum to one across two ideal types (see Table 2). These countries differ only on one crucial dimension—leave duration—from belonging to another model. Sweden is divided between the supported universal breadwinner (0.59) and universal caregiver (0.41) model, and indeed several authors have already noticed that Swedish policies go further than other European countries in promoting father’s active engagement in childcare (Ellingsæter 2014). Ireland, Italy, the Netherlands and the United Kingdom present instead scores that split evenly between the male breadwinner (MB) and the unsupported universal breadwinner (UUB) model (Table 2). This also serves to illustrate the close relationship between those two models. While the MB has explicit policies in place to reinforce the traditional family model, the UUB residual approach supports this model implicitly. Minimalist provisions such as those of the UUB do not alter the state of existing inequalities, thus perpetuating prevailing norms about women’s primacy in caregiving. Accordingly, these ideal types hold similar ideas with regard to maternal responsibility for childcare, but differ with regard to the extent of public intervention in the family sphere.

#### Indirect approach

The second approach to the analysis hybrids is closely related to the previous and consists in experimenting with different *thresholds* concerning key dimensions. The choice of an alternative threshold does not necessarily invalidate the original analysis, but rather acknowledges that its value can in some cases be strongly contextual. For instance, the optimal length of parental leave is rather contested and reflects considerations about maternal employment, parental right to time to perform childcare and children’s right to spend time with their parents. This value is likely to vary across countries and to be influenced by other institutions and policies (e.g. availability of flexible working-time, occupational structures, other childcare provisions) (Gornick and Meyers 2009). In their analysis, Ciccia and Verloo used an optimal duration of 78 weeks per family. Here, a lower value of 52 weeks was chosen (considered optimal, for instance, in Gornick and Meyers 2009, Javornick 2014).^1^ The fuzzy scores for leave duration with the 52 weeks threshold and the resulting membership scores are reported in Table 3.

**Table 3:** Fuzzy membership scores of parental leave policies (parental leave duration thresholds: 156, 52, 14), 2012

Country	Fuzzy scores leave duration (52 weeks)	Universal careeiver (FUC)	Limited universal caregiver (LUC)	Supported universal breadwinner (SUB)	Unsupported universal bread winner (UUB)	Careeiver paritv (CGP)	Male breadwinner
Austria	0.82	0.05	0.05	0.05	0.18	0.05	0.82
Belgium	0.31	0.21	0.21	0.21	0.60	0.21	0.31
Bulgaria	0.83	0.06	0.06	0.17	0.17	0.83	0.17
Cyprus	0.35	0.01	0.01	0.01	0.65	0.01	0.35
Czech Republic	1.00	0.00	0.00	0.00	0.00	0.00	0.94
Denmark	0.50	0.12	0.12	0.50	0.05	0.50	0.05
Estomia	0.96	0.05	0.04	0.04	0.04	0.18	0.79
European Union	0.44	0.00	0.00	0.00	0.56	0.00	0.44
Finland	0.48	0.48	0.52	0.05	0.05	0.05	0.05
France	1.00	0.00	0.00	0.00	0.00	0.00	0.87
Germany	0.97	0.01	0.01	0.01	0.03	0.01	0.07
Greece	0.42	0.03	0.03	0.03	0.58	0.03	0.42
Hungary	0.96	0.07	0.04	0.04	0.04	0.35	0.65
Iceland	0.26	0.26	0.74	0.01	0.01	0.01	0.01
**Ireland**	0.63	0.03	0.03	0.03	**0.37**	0.03	**0.63**
**Italy**	0.62	0.06	0.06	0.14	**0.38**	0.14	**0.62**
Latvia	0.97	0.01	0.01	0.01	0.03	0.01	0.66
Lithuania	0.97	0.26	0.03	0.03	0.03	0.58	0.33
Luxembourg	0.61	0.09	0.09	0.33	0.29	0.33	0.29
Malta	0.30	0.02	0.02	0.02	0.70	0.02	0.30
**Netherlands**	0.61	0.11	0.11	0.11	**0.39**	0.11	**0.61**
Norway	0.42	0.42	0.52	0.00	0.00	0.00	0.00
Poland	0.97	0.00	0.00	0.00	0.03	0.00	0.91
Portugal	0.48	0.48	0.52	0.00	0.00	0.00	0.00
Romania	0.89	0.07	0.07	0.11	0.08	0.84	0.08
Slovakia	0.96	0.01	0.01	0.01	0.04	0.01	0.84
**Slovenia**	0.59	0.15	0.15	**0.41**	0.09	**0.59**	0.09
Spain	1.00	0.00	0.00	0.00	0.00	0.00	0.79
**Sweden**	0.63	**0.63**	**0.37**	0.00	0.00	0.00	0.00
Switzerland	0.05	0.05	0.05	0.88	0.12	0.05	0.05
United Kingdom	0.69	0.00	0.00	0.00	**0.31**	0.00	**0.69**

Scores in bold indicate case that have changed membership using the 52 weeks intermediate threshold for leave duration

This leads to substantially similar results but for five countries. Italy, Ireland and the Netherlands move from the UUB to the MB model, while Sweden (.63) and Slovenia (.59) are now classified respectively as universal caregiver and caregiver parity model. This essentially confirms the results obtained with the first approach, but also allows to detect Slovenia as a hybrid of universal breadwinner and caregiver parity model. In particular, this hybrid form points at the limitations of policies that prioritize women employment as the criteria for gender equality. In the supported universal breadwinner model, first the women then the state have responsibility for childcare, while fathers’ role remains neglected perpetuating inequalities in the domestic division of labour. Indeed, there are few incentives for fathers to use parental leave in Slovenia and only 15 days of paternity leave are fully paid. As a result, 80 percent of fathers used the fully-paid leave in 2009, but only 15 percent took more than 15 days and just around 6 percent took any of the shared leave (Moss 2011).

#### Combinatory approach

The final method to study hybrids consists in exploiting the whole property space, i.e. computing fuzzy scores for all logically possible combination of dimensions. The number of possible types is 2^k^, with K equal to the number of dimensions considered. The guiding principles for applying this procedure are that those hybrid types should be both theoretically meaningful and empirically not empty.^2^ Since cases can achieve membership only in one type, this method can only limitedly be used here. However, we can observe that the limited universal caregiver type identified by Ciccia and Verloo would be considered a hybrid using this method, which combines elements of the universal caregiver (incentives for fathers to use leave) and of the universal breadwinner (relatively short duration to promote a rapid re-entry into employment) types. This procedure is also be used here to analyse Germany, which remained unclassified in Ciccia and Verloo (2012). In formal terms, the German parental leave configuration (T*m*G*F) differs only for one element (F or father’s incentives to use leave) from the male breadwinner (T*m*G*f).^3^ This indicates that in order to understand this case, we need to focus on developments concerning this element. The introduction of an earnings-related sharing bonus in 2007 has represented a paradigmatic shift in German childcare policy traditionally supporting the male breadwinner model. Nonetheless, this change remains highly conflictual and has resulted in many policy inconsistencies such as the introduction of a benefit for stay-at-home parents, or the fact that paternity leave comes in the form of a sharing-bonus which perpetuates the idea of the primacy of women in caregiving. Thus, the German model is definitely ‘on the move’, but has not totally shed its male breadwinner legacy (Morgan 2013). This hybrid form is produced by the co-existence of competing visions of the family, which is also evident in the implementation of new legislation on the expansion of childcare services. The federal structure of the German state has allowed for considerable variation with regard to levels of provisions at the regional level in spite of the partisan convergence at the national level over the need of a more ‘social democratic’ childcare policy (Andronescu and Carnes 2015).

This section demonstrated three methods to identify specific hybrid forms arising from tensions in the underlying policy rationale. In the next section, I move on to consider a further potential source of hybridization deriving from the ways in which policies relate to one another.

## Step 2: aggregating policy analyses into a typology of childcare policies

Hybrids welfare form are not only produced by tensions within policies as highlighted in step 1, but also by the ways in which policies interconnect within an overall policy package. Therefore, in step 2 the analysis moves on to consider the relationship between different childcare policies by carrying out an aggregation procedure of the results of analyses of policies for parental leave (Ciccia and Verloo 2012), and ECEC services^4^ in thirty European economies. This analysis produces an overall typology of childcare policies which clearly distinguishes between pure, hybrid and sub-types, thus, emphasizing differences in the ways policies combine and interact with each other.

In order to aggregate childcare policies, this paper draws on the fuzzy scores of ECEC policies used in Ciccia and Bleijenbergh (2014). The authors considered four dimensions in their analysis: (1) childcare coverage (S); (2) childcare mix (C); (3) formal childcare time (H); (4) public financial support for childcare (M) (see Table 1 for the main characteristics of ECEC ideal types). Table 10 in the appendix describes the measures and thresholds that were used to calculate countries’ fuzzy scores on those dimensions (Appenix 1, table 11). Table 12 reports the configuration of the sets with regard to the ECEC ideal types.

While previous analyses by Ciccia and Verloo (2012) and Ciccia and Bleijnebergh (2014) focused on classifying countries by analysing single policy measures, this paper proposes an approach for the systematic analysis of the complexity inherent policy packages. The assessment of the overall childcare regime would require the incorporation of many other policies—in particular, working time regulation, tax credits, care allowances, and regulatory policies towards childcare providers—however, previous research has foremost focused on these two policies because they more clearly reflect the normative script for socially acceptable gender division of roles and allocation of childcare (Javornik 2014). Moreover, the aim of this analysis is mainly to illustrate how typologies can be built to describe the plurality of models underlying national childcare configurations. Indeed, the proposed typology demonstrates that many countries combine childcare policies with different underlying gender norms and gender equality ideas.

The aggregation procedure is carried out through the principle of set-intersection, which was already used to combine policy dimensions at the level of policy analysis, i.e. the conformity of a case to a given type is given by the minimum value score in the set formed by the two policy configurations. For instance, a case scoring 0.80 on the male breadwinner leave type and 0.90 on the male breadwinner ECEC type would be fully allocated within the male breadwinner childcare policy type (0.80). Depending on the way in which policies bind together, we can have different amalgams: (1) *pure types*, when both policies endorse the same principle (membership scores >0.50 on the same ideal type for both policies); (2) *hybrid types* in which different principles and models coexist (membership scores >0.50 on different ideal types); (3) *sub*-*types*, i.e. types showing that similar principles may be enforced through different policy designs (membership scores >0.50 on similar ideal types). The aggregate fuzzy membership scores are reported in Tables 4, 5, 6. This analysis excludes four countries (Belgium, Germany, Luxemburg, and the Netherlands) that remained unclassified in the original analyses.

**Table 4:** Aggregate fuzzy membership scores of childcare policy pure types

Male breadwinner	Caregiver Parity	Unsupported universal breadwinner	Supported universal breadwinner
Austria (0.57)	Bulgaria (0.60)	Malta (0.54)	Denmark (0.73)
Czech Republic (0.57)	Lithuania (0.58)	Ireland (0.57)	
Latvia (0.51)	Romania (0.71)	Italy (0.60)	
Poland (0.91)		Cyprus (0.83)	
Slovakia (0.84)

**Table 5:** Aggregate fuzzy membership scores of childcare policy hybrid types

Limited universal caregiver/caregiver parity	Limited universal caregiver/unsupported universal breadwinner	Limited universal caregiver/supported universal breadwinner
Finland (0.56)	Portugal (0.50)	Sweden (0.57)
Norway (0.57)		Iceland (0.59)

**Table 6:** Aggregate fuzzy membership scores of childcare policies sub-types

Male breadwinner/one-and-a-half breadwinner	Supported universal breadwinner/one-and-a-half breadwinner	Male breadwinner/unsupported universal breadwinner	Unsupported universal breadwinner/male breadwinner	Male Breadwinner/caregiver parity
United Kingdom (0.71)	Switzerland (0.52)	France (0.56)	Greece (0.80)	Estonia (0.68)
		Spain (0.57)		Hungary (0.63)

## Pure types

Table 4 presents results with regard to thirteen countries (50 percent of cases) that are consistently classified in the same ideal type. These are pure types with regard to their childcare policy. In these countries, parental leave and ECEC policies endorse the same underlying ideal. It may be observed that a number of countries show low memberships (0.50–0.60). This derives from the choice of the stricter aggregation criteria (strong intersection), which assumes that these policies cannot be tradedoff against each other.

This choice derives from theoretical understandings that ECEC and parental leave policies are equally important in shaping parental usage of childcare policies, i.e. they are ‘bundles of inseparable provisions’(Gornick and Meyers 2003). This issue could become problematic when incorporating a high number of policies since membership scores will tend to become low. To obviate this problem, it is also possible to use other aggregation functions depending on the theoretical meaning that we attribute to the relation between the policies considered.^5^

### Hybrid types

The remaining countries present membership scores for parental leave and ECEC services that fall within different ideal types. While the idea of a one-to-one correspondence between policies is implicit in many analyses, the co-existence of different types does not necessarily imply that their childcare policy lacks an overall logic or that it ‘does not make sense’, rather the allocation of these countries to a type is a matter of theoretical and substantial interpretation of different combinations of ideal types.

Five countries (Sweden, Iceland, Finland, Norway and Portugal) combine limited universal caregiver (LUC) leaves, supporting a balanced gender division of childcare, with different types of ECEC provisions (Table 5). These countries are hybrids since their childcare policies contain to some extent contradictory principles. Sweden and Iceland combine LUC leave policies with supported universal breadwinner ECEC services (i.e. universally accessible, full-time, publicly financed) intended to support maternal employment. While these countries have achieved substantial gender equality goals, two principles of their welfare states limit the achievement of a universal caregiver model. First, public responsibility for formal childcare is a long-standing principle in these countries, and there is a long normative tradition that children are better educated in full-time public childcare from around 1 year. Secondly, these countries are strongly focused on maintaining high employment and childcare policies are designed to serve this overarching goal. Thus, for instance, parental leave regulations strongly incentivize parents to return to work within a year of childbirth. While these principles positively emphasize individuals’ right not to be full-time carers, they also restrict the recognition of the right to time for childcare, which represents an important principle of the universal caregiver model. The full implementation of such ideal would require a fundamental restructuring of paid employment, and in particular of full-time work (e.g. through incentives for both mothers and fathers to use reduced working hours and part-time ECEC facilities) (Crompton 1999).

Finland and Norway are instead classified as limited universal caregiver/caregiver parity type. Their policies show the presence of tensions between the aims of promoting gender equality and supporting a traditional family model with the mother as the main care provider. Despite sharing the Nordic universalistic approach to childcare services, the volume of day-care differs substantially from the other Nordic countries. Norway is a latecomer with regard to the right to a place in childcare services, which was introduced only in 2009. In Finland the expansion of services has been severely hampered by the provision of a cash-for-care scheme supporting parental care in the home. Important differences also concern policies to promote a more active role of fathers in childcare. A father’s quota was introduced in Finland only in 2013 (previously, a sharing bonus), while the recently elected centre-right government in Norway has reduced the daddy quota from fourteen to ten weeks, and proposed new rules to make this period more easily transferable to the mother. Hybridisation in these countries points at the presence of ongoing political divisions over competing childcare visions, which is especially likely to persist over the two policy measures—daddy quotas and cash-for-care benefits—where these values are put in play (Ellingsæter 2014). Finally, Portugal is classified as a limited universal caregiver/unsupported universal breadwinner type because of limited public investment in childcare services. In spite of the steady increase in recent years, the country childcare system continues to rely heavily on family-based and informal solutions (e.g. grandparents, child-minders), while formal day care is mainly provided by non-profit organizations (e.g. the Catholic Church) (Tavora 2012). The rejection of the traditional family model shows more clearly in the leave policy design with the introduction in 2009 of individual entitlements, a daddy quota and significant incentives for flexible uptake, which are intended to promote a more equal sharing of parental leave.

### Sub-types of childcare policies

Policies can align to the same principle (pure types) or to some extent contradict each other (hybrids). A third possible combination occurs when different type policies connect in ways that end up endorsing the same or similar principles. Likewise the case of policy dimensions in the analysis of single policies, the configurational principle entails that the meaning of policies is relational to the whole policy configuration. In these countries, the way in which policy types combine does not reflect differences with regard to the underlying concept of the typology–the role of men and women in paid and care work–but rather in some other principle (e.g. the role of the state or the welfare of children). In this case, policies work in the same direction, but differ in design because they reflect differences in those other principles and aims.

In our case, the United Kingdom and Switzerland are characterized respectively as male breadwinner (0.71) and unsupported (0.52) one-and-a-half breadwinner models, while Greece, France and Spain present different combinations of male breadwinner and unsupported universal breadwinner policies (Table 6). The similarity between the unsupported universal breadwinner (UUB) and male breadwinner (MB) model have already been remarked on in Sect. 5. The one-and-a-half breadwinner model is also generally considered a modification rather than a transformation of the MB model since it is not associated with substantial changes in gender relations, nor does it guarantee women with real financially autonomy (Crompton 1999). Accordingly, these countries represent subtypes of the male breadwinner model rather than hybrids because they uphold similar gender norms. For instance, Greece providing for short low-paid leaves (UUB) represents an even more residual version of the male breadwinner model. France and Spain provide more ECEC provisions than other pure MB countries, but the overall level of support for children 0–2 remains low. Similarly, Hungary combining MB leave and caregiver parity ECEC policies represent a less generous sub-type of the caregiver parity model, which is more focused on children than on rewarding mothers. These policy configurations show that similar gender ideals can be upheld through different measures, although these differences may still reflect variation in other principles such as the extent of the state intervention in the family sphere or the investment in children.

## Conclusions

The presence of hybrids and policy heterogeneity in many welfare states have led a growing number of scholars to question the usefulness of typologies and to doubt the idea that welfare states represent coherent wholes of any sort (Bannink and Hoogenboom 2007; Kasza 2002). In this paper, I propose a two-step FSITA approach to deal with these issues and contribute to the systematic analysis of hybrids at the level of both policies and policy configurations. The first step focus on single policies to identify specific hybrid forms arising from tensions in their underlying rationale. While previous studies have most frequently focused on partial memberships, this procedure shows that our understanding of policy hybrids can also be informed by the analysis of non-membership scores, the underlying dimensions of the typology and the investigation of the configurational space. The second step focused on the relation between policies and develops a typology of childcare policies by carrying out an aggregation procedure of parental leave and ECEC policies. This analysis shows that patterns of social provision exist, but that they are not necessarily congruent and that policies interconnect in different ways. They can map into each other (pure types), they can adopt similar principles but differ in programme design (sub-types), or even endorse principles that partly contradict each other (hybrids). Some of the reasons this occurs derive from processes of reforms which are not quite accomplished (Morgan 2013), partisan conflict (Ellingsæter 2014) or the existence of different norms and systems at the sub-national level (Andronescu and Carnes 2015).

This two-step approach has important implications for the comparative analysis of welfare states. First, this approach reflects more closely current understandings of welfare states as complex and not necessarily congruent objects and draws the attention to the co-existence of multiple models and the importance of theoretical and substantial interpretation of policy configurations in order to make sense of those complexities. Secondly, it demonstrates that disaggregated and aggregated analyses are not alternative, rather they are equally important to account for hybrid welfare forms. While disaggregated analyses are necessary to account for the multidimensionality of social policies, aggregated analyses enhance our understanding of policy interactions and countervailing (as well as reinforcing) effects between policies.

A clearer understanding of hybrids can also bear important implications for the understanding of processes of policy change. According to Crouch (2001), hybrid welfare states point at a menu of inherited characteristics and policy legacies, which can be exploited by social and political actors to introduce innovative change when the dominant strand is no longer considered useful to resolve a particular set of problems. In this view, the plurality of forms within welfare states could be viewed as a set of possible likely futures, which can be used to generate hypotheses about the direction of policy reforms. The study of how policy actors shape and engage with these options represent a promising area for future research.

## Appendix 1

See Tables 7, 8, 9, 10, 11, 12.

**Table 7:** Dimensions measures and thresholds for parental leave policies

Dimensions	Measures	Thresholds		
		1.00	0.50	0.00
Leave duration (L)	Total number of weeks of leave per family	156	78	14
Monetary compensation (M)	Full-time equivalent (FTE) leave*/total leave time per family	0.90	0.60	0.40
Distribution of entitlements (G)	1–(FTE mother’s leave–FTE father’s leave)/(FTE mother’s leave + FTE father’s leave)	0.00	0.60	0.85
Incentives for fathers (F)	Index of fatherhood opportunity	16	9	0

FTE leave is calculated as the duration of paid weeks of leave multiplied by the wage replacement rate

**Table 8:** Fuzzy scores for parental leave policies

Country	Duration (T)	Monetary compensation (M)	Distribution of entitlements (G)	Father’s incentives (F)
Austria	0.73	0.05	0.95	0.09
Belgium	0.16	0.21	0.60	0.21
Bulgaria	0.75	0.83	0.94	0.12
Cyprus	0.17	0.01	0.95	0.06
Czech Republic	1.00	0.00	0.95	0.06
Denmark	0.23	0.95	0.88	0.27
Estonia	0.97	0.18	0.95	0.21
Finland	0.22	0.83	0.44	0.95
France	1.00	0.00	0.87	0.12
Germany	0.97	0.01	0.52	0.93
Greece	0.20	0.03	0.94	0.06
Hungary	0.96	0.35	0.93	0.21
Iceland	0.14	0.88	0.01	0.95
Ireland	0.41	0.03	0.95	0.06
Italy	0.40	0.14	0.66	0.06
Latvia	0.98	0.01	0.91	0.34
Lithuania	0.97	0.67	0.74	0.42
Luxembourg	0.39	0.71	0.33	0.09
Malta	0.15	0.02	0.94	0.12
Netherlands	0.39	0.11	0.62	0.12
Norway	0.20	0.97	0.00	0.89
Poland	0.98	0.00	0.93	0.09
Portugal	0.22	0.53	0.00	0.85
Romania	0.85	0.92	0.93	0.16
Slovakia	0.96	0.01	0.95	0.16
Slovenia	0.35	0.91	0.85	0.34
Spain	1.00	0.00	0.86	0.21
Sweden	0.41	0.73	0.00	0.95
Switzerland	0.05	0.88	0.95	0.05
United Kingdom	0.52	0.00	0.93	0.06

Based on Ciccia and Verloo (2012)

**Table 9:** Property space of ideal typical configurations of parental leave policies

Ideal type	Leave duration	Monetary compensation	Distribution of entitlements	Incentives for fathers
Male breadwinner	L	m	G	f
Caregiver parity	L	M	G	f
Unsupported universal breadwinner	l	M	G	f
Supported universal breadwinner	l	M	G	f
Limited universal caregiver	l	M	g	f
Full universal caregiver	L	M	g	F

Uppercase letters indicate membership in a set; lowercase letters denote the absence or negation of the set

**Table 10:** Dimensions measures and thresholds for ECEC policies

Dimensions	Measures	Thresholds		
		1.00	0.50	0. 00
Childcare coverage (S)	Effective childcare coverage rate (0–2 years)	125	52	31
Childcare mix (C)	Childcare mix index (0–2 years)	80	50	20
Formal childcare time(H)	Average number of weekly hours in formal care (0–2 years)	30	20	10
Public financial support (M)	Public expenditure on family services and pre-primary education as percentage of GDP adjusted for the proportion of children 0–5 years	0.40	0.30	0.10

**Table 11:** Fuzzy scores for ECEC policies

Country	Effective childcare coverage (S)	Childcare mix (C)	Childcare hours (H)	Public financial support (M)
Austria	0.06	0.02	0.57	0.18
Belgium	0.52	0.92	0.94	0.18
Bulgaria	0.60	0.04	0.99	0.29
Cyprus	0.05	0.90	0.98	0.06
Czech Republic	0.43	0.01	0.35	0.10
Denmark	0.89	0.89	0.99	1.00
Estonia	0.68	0.07	0.99	0.05
Finland	0.60	0.32	0.99	0.71
France	0.44	0.86	0.94	0.23
Germany	0.49	0.18	0.82	0.50
Greece	0.05	0.14	0.97	0.05
Hungary	0.63	0.02	0.97	0.71
Iceland	0.57	0.83	0.99	0.94
Ireland	0.12	0.65	0.57	0.02
Italy	0.33	0.62	0.95	0.23
Latvia	0.49	0.13	1.00	0.23
Lithuania	0.79	0.02	1.00	0.23
Luxembourg	0.56	0.45	0.89	0.07
Malta	0.01	0.54	0.57	0.18
Netherlands	0.63	0.87	0.23	0.14
Norway	0.70	0.43	0.97	0.71
Poland	0.03	0.02	0.99	0.05
Portugal	0.50	0.76	1.00	0.16
Romania	0.71	0.02	0.82	0.21
Slovakia	0.04	0.02	0.94	0.07
Slovenia	0.62	0.39	0.99	0.26
Spain	0.43	0.94	0.86	0.39
Sweden	0.83	0.65	0.94	0.92
Switzerland	0.07	0.95	0.29	0.05
United Kingdom	0.20	0.95	0.14	0.08

Based on Ciccia and Bleijenbergh (2014)

**Table 12:** Ideal typical configurations of ECEC policies

Ideal type	Childcare coverage (S)	Childcare mix (C)	Formal childcare time (H)	Public financial support (M)
Male breadwinner	s	c	h or H	m
One-and-a-half-breadwinner	s	C	h	m
Caregiver parity	S	c	H	m or M
Unsupported universal breadwinner	s	C	H	m
Supported universal breadwinner	S	C	H	M
Full universal caregiver	S	C	h	M

Uppercase letters indicate membership in a set; lowercase letters denote the absence or negation of the set

## Footnotes

1 These entitlements might appear already very generous in other regional contexts (e.g. the United States), but in Europe the Maternity Leave (92/85/EEC), and Parental Leave Directives (2010/18/EU) already establishes a joint minimum floor of over 15 months per family.

2 Hudson and Kuhner (2010) used this method to classify welfare states on the basis of four protective and productive dimensions of welfare states, which combined into a total of sixteen types. Four were considered ‘pure’ types, while the remaining twelve were also considered relevant to understand the interaction of productive and protective functions of social policy.

3 Where uppercase letters indicate membership in a set, lowercase letters the absence or negation of the set, and * = logical AND or the intersection of the sets.

4 ECEC services refer to education at pre-school or equivalent, education at compulsory education, childcare at centre-based services outside school hours, or childcare at day-centre organized/controlled by a public or private structure.

5 For instance, the union function (maximum score) could be used if we imagined that policies substitute for each other (e.g. if parents chose indifferently between leaves and ECEC services) or weak intersections (algebraic product) could be used if we allowed for the possibility of compensation effect between policies (e.g. working time regulations could compensate for meagre leave provisions). Since parents are not indifferent between parental leave and ECEC, this was not the case in this analysis, and both policies are needed to configure the various ideal types.
